# Supplementary material for: Retrospective Evaluation of Clinical and Clinicopathologic Findings, Case Management, and Outcome for Dogs and Cats Exposed to Micrurus fulvius (Eastern Coral Snake): 92 Cases (2021–2022)
Source: Toxins (Basel). 2024 May 27;16(6):246. doi: 10.3390/toxins16060246 (PMC11209501; doi:10.3390/toxins16060246)
Supplement: Supplementary file 1 [file toxins-16-00246-s001.zip › toxins-2981174-supplementary.pdf]

toxins

MDPI

Retrospective Evaluation of Clinical and Clinicopathologic Findings, Case Management, and Outcome for Dogs and Cats Exposed to *Micrurus fulvius* (Eastern Coral Snake): 92 Cases (2021–2022)

Table S1. Demographic data for dogs (sex and breed). Dog breeds that were represented only once are listed as "other". All cats were male neutered Domestic Shorthairs.

Characteristic	Dogs (n=83)
**Sex**	
MC	33(39.8%)
MI	9 (10.8%)
F.S.	35 (42.2%)
F.I.	7 (8.4%)
**Breed**	
Mixed Breed	29
Labrador Retriever	7
German Shorthaired Pointer	4
Boxer	3
French Bulldog	3
Jack Russell Terrier	3
Dachshunds	3
Australian Cattle Dog	3
Australian Shepherd	2
Doberman	2
Weimaraner	2
Persa Carino	2
Yorkshire Terrier	2
German Shepherd	2
Other	16
